# Cardiac magnetic resonance assessment of cardiac involvement in autoimmune diseases

**DOI:** 10.3389/fcvm.2023.1215907

**Published:** 2023-09-22

**Authors:** Avanti Gulhane, Karen Ordovas

**Affiliations:** Department of Radiology, University of Washington, School of Medicine, Seattle, WA, United States

**Keywords:** autoimmune, rheumatic, cardiac magnetic resonance, biomarkers, prognostication, inflammation, microvasculopathy, cardiotoxicity

## Abstract

Cardiac magnetic resonance (CMR) is emerging as the modality of choice to assess early cardiovascular involvement in patients with autoimmune rheumatic diseases (ARDs) that often has a silent presentation and may lead to changes in management. Besides being reproducible and accurate for functional and volumetric assessment, the strength of CMR is its unique ability to perform myocardial tissue characterization that allows the identification of inflammation, edema, and fibrosis. Several CMR biomarkers may provide prognostic information on the severity and progression of cardiovascular involvement in patients with ARDs. In addition, CMR may add value in assessing treatment response and identification of cardiotoxicity related to therapy with immunomodulators that are commonly used to treat these conditions. In this review, we aim to discuss the following objectives:

• Illustrate imaging findings of multi-parametric CMR approach in the diagnosis of cardiovascular involvement in various ARDs;
• Review the CMR signatures for risk stratification, prognostication, and guiding treatment strategies in ARDs;
• Describe the utility of routine and advanced CMR sequences in identifying cardiotoxicity related to immunomodulators and disease-modifying agents in ARDs;
• Discuss the limitations of CMR, recent advances, current research gaps, and potential future developments in the field.

## 1. Introduction

High burden of cardiovascular disease (CVD) and associated morbidity and mortality exists in various autoimmune rheumatic diseases (ARDs). These include rheumatoid arthritis (RA), Systemic lupus erythematosus (SLE), spondyloarthritides such as psoriatic arthritis (PsA) and ankylosing spondylitis (AS), systemic sclerosis (SSc), Sjögren's syndrome (SS), mixed connective tissue disease (MCTD), inflammatory myopathies such as dermatomyositis (DM), polymyositis (PM) and inclusion body myositis (IBM) and vasculitis of large, medium and small vessels (1–5). Life expectancy is low in patients with ARDs compared to the general population (6) mainly due to increased and often subclinical prevalence of CVD despite the introduction of new and targeted treatment strategies to reduce disease-related mortality (5, 7–9). Traditional imaging techniques are not well equipped to define the acuity of cardiac involvement in ARDs due to limited tissue characterization abilities (10–13). This particular limitation on routinely performed transthoracic echocardiography (TTE) leads to under-diagnosis of early myocardial involvement that may lead to fatal arrhythmias despite preserved LV and RV function (14).

Cardiac magnetic resonance (CMR) is a non-invasive examination with diagnostic as well as prognostic capabilities with cardiovascular involvement in patients with ARDs. The various pathophysiologic processes that can be accurately assessed on CMR include myocardial inflammation (10, 15, 16) early or late fibrosis due to inflammation (17), macro- and micro-vasculopathy (18, 19), ischemic heart disease, and myocardial infarction(18, 20, 21), rhythm and conduction disturbances, valvular diseases, pulmonary hypertension, and diastolic or systolic heart failure (3, 22). In particular, high accuracy of stress CMR is beneficial for early assessment of ischemic or hibernating myocardium resulting from epicardial coronary artery diseases in patients with ARDs (21). Techniques for vessel wall imaging can help identify arterial wall inflammation of the great vessels and overall active inflammatory burden in ARDs (20).

As per the recent clinical consensus document by the European Association of Cardiovascular Imaging (EACVI), CMR should be obtained for clinical evaluation of patients with cardiac symptoms when the echocardiogram is normal since the disease processes can be occult to echocardiogram. CMR should be considered as first line imaging modality to assess patients with SSc or SLE if there is any suspicion of cardiac involvement at the time of the diagnosis. Finally, CMR should be the next test to perform a detailed evaluation of the extent of myocardial and vascular disease burden in patients with ARD with cardiac symptoms and abnormal echocardiogram (23). CMR is the test of choice for monitoring treatment response when there is cardiac involvement and allows identification of cardiotoxicity in these patients who are often treated with long-term immunomodulatory therapies (24).

Imagers and referring providers must have a robust knowledge of the multi-parametric CMR approach for the appropriate management of ARDs. Hence, in this review, we will focus on how CMR helps in the well-rounded assessment and management of various cardiovascular manifestations in patients with ARDs.

## 2. Multiparametric CMR approach for diagnosing cardiac involvement in various ARDs

A comprehensive CMR examination for patients with ARDs consists of T1-weighted (T1w) and T2-weighted (T2w) inversion-recovery turbo spin-echo (black blood) images or STIR-T2 images multiplanar balanced steady-state free precision (bSSFP) bright blood cine images,, look locker and phase-sensitive inversion recovery (PSIR) sequence for early and late gadolinium enhancement (LGE), modified look locker technique for T1 and T2 parametric mapping, post contrast T1 mapping for ECV quantification. Newer techniques such as strain may be used for the early detection of regional function abnormalities. Quantitative perfusion mapping should be considered when available (25). Rich multi-parametric data provided through a single CMR examination allows for capturing the intricate details of various disease processes in ARDs.

### 2.1. Myocardial inflammation first-line

CMR allows the identification of myocardial edema from acute myocarditis on STIR- T2w black blood images. Native T1 and T2 parametric mapping are popular techniques for objective analysis and quantification of myocardial fibrosis and inflammation. Native T1 mapping can be elevated in the early stages of disease, when inflammation is the main abnormality, but can also be at late stages when only fibrosis is seen. Therefore, the combined assessment with both native T1 and T2 maps is quite helpful to differentiate edema from fibrosis. Extracellular volume (ECV) estimates the expansion of the extracellular space due to edema or fibrosis (26, 27). In both acute and chronic myocarditis, the most characteristic location of myocardial edema on CMR is at the subepicardial basal to mid inferolateral segment in a noncoronary distribution on T2w and LGE images (28). In our clinical experience, myocardial edema in ARD affects the myocardium globally and changes are much more subtle than in classic myocarditis.

In patients with ARDs, signs of myocardial involvement are infrequent in the clinical setting but extremely common in autopsy studies. Several studies report the presence of myocardial inflammation in up to 40%–50% of patients of RA (29) and SLE (30, 31) and in around 40% of patients in SS (32, 33), PM, and DM (34). Myocarditis is extremely rare in Sjogren's syndrome (35). In a small cohort of 20 patients with different ARDs, myocarditis was identified in most patients (80%) using CMR with a cut-off of 1.89 ± 0.25 for the signal ratio of the myocardium to skeletal muscle on T2w images, relative myocardial enhancement of 11.31 ± 11.18 on T1w images and epicardial late gadolinium enhancement. This cut-off had a higher agreement with histology and PCR (50% and 80% of positive CMR examinations) respectively (10).

**Figure 1:**
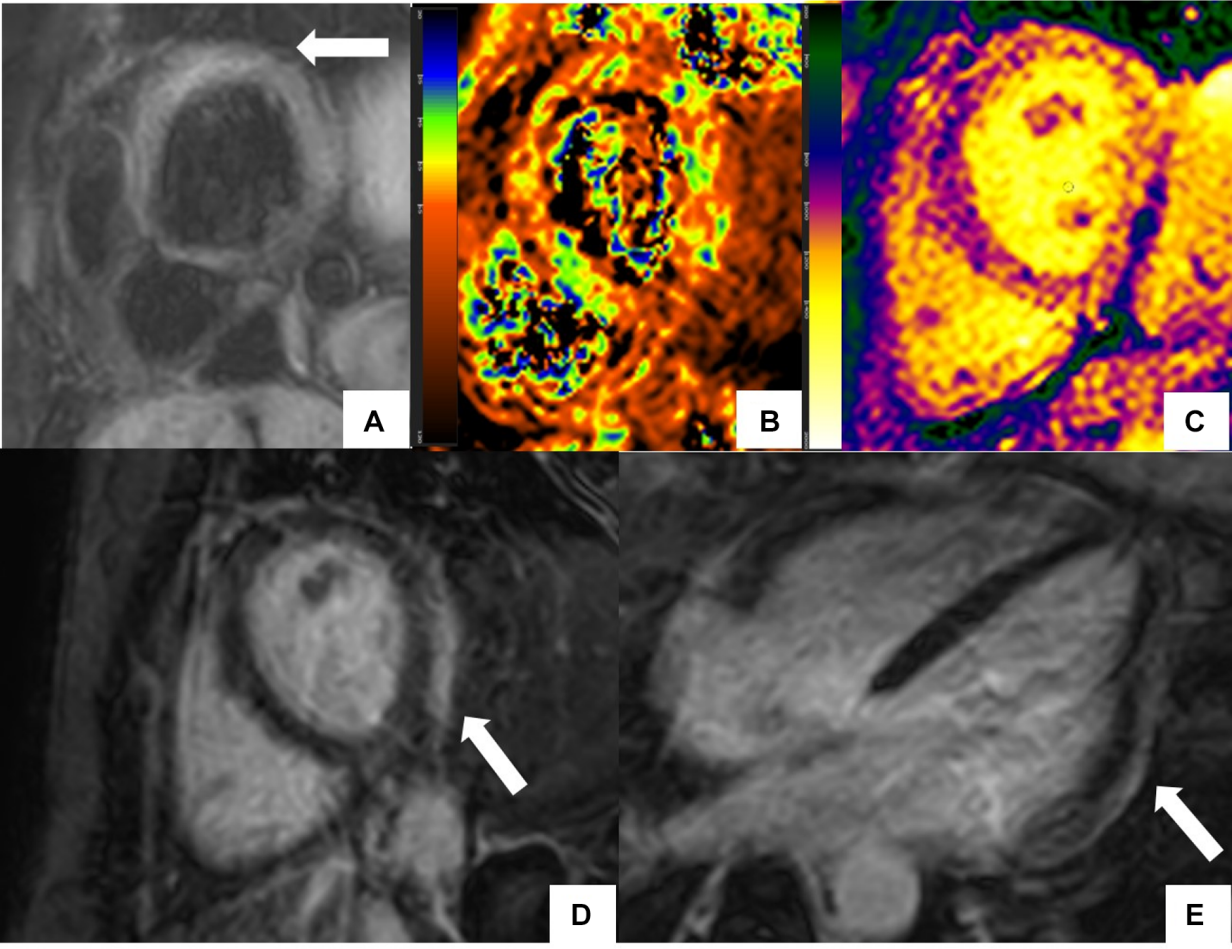
CMR findings in systemic lupus erythematosus (SLE) myopericarditis. 44 year old female with longstanding systemic lupus erythematosus (SLE) and overlap syndrome with premature ventricular contractions and dyspnea on exertion. Cardiac MR (CMR) showed mild biventricular systolic dysfunction with left ventricular ejection fraction (LVEF) of 48% and right ventricular ejection fraction of (RVEF) of 42%. Edema was seen on T2 black blood fat supressed short axis image (**A**) with elevated T2 value of 72 ms on T2 color map (**B**) and diffusely increased myocardial native T1 value of 1,134 ms on native T1 color map (**C**) On short-axis and 4-chamber late gadolinium enhancement phase-sensitive inversion recovery (LGE- PSIR) image, subepicardial hyperenhancement was seen within the entire inferolateral wall (**D,E** respectively). Constellation of findings were consistent with cardiac involvement in SLE with an acute inflammatory component (42). Pericardial thickening with diffuse LGE and increased T2 signal in the basal anterior wall away from LGE suggested acute on chronic pericarditis. No pericardial effusion was seen.

In patients with SLE, disease activity has been traditionally evaluated using the European Consensus Lupus Activity Measurement (ECLAM) index (36) and the newer SLE Disease Activity Score (SLE-DAS) (37). CMR indices such as relative T2 ratio, prolonged myocardial T1 relaxation time, global relative enhancement derived from a subtracted image (38), and focal subepicardial or mid myocardial LGE in the inferolateral wall have been reported to correlate with disease activity assessed by ECLAM index (39) (Figure 1). T2 relaxation values are a sensitive indicator of myocardial disease in patients with active or subclinical SLE and may normalize with clinical improvement in myocardial disease activity (40). T2w images also help with the characterization of acute vs. chronic myocardial involvement using simple ratios such as T2 signal intensity ratio > 2 relative to skeletal muscle for acute lesions with myocardium in patients with inflammatory vasculitis (41).

The updated Lake Louise criteria for myocarditis incorporates at least one T2-based criterion such as a global or regional increase of myocardial T2 relaxation time or an increased signal intensity in T2w CMR images, with at least one T1-based criterion such as increased myocardial T1, ECV, or LGE for increased specificity and diagnostic accuracy to detect acute myocardial inflammation. Its supportive criteria include CMR-based identification of pericarditis and systolic LV dysfunction in the form of global or regional wall motion abnormality on cine imaging (27). CMR has a high sensitivity of 76%, a specificity of 95.5%, and a diagnostic accuracy of up to 85% when a combined approach using T2w and early and late gadolinium enhancement is employed for the detection of myocardial inflammation (10, 41, 43). T2 mapping seems to perform better in characterizing both acute and chronic myocarditis than T1 mapping (44). Parametric T2 mapping is also independent of concomitant skeletal muscle inflammation. It provides more robust information on myocardial edema compared to T2W ratio due to reduced sensitivity to motion artifacts from motion correction and clear delineation of endocardial borders, increased objectivity from pixel-wise information of native T2 value and reduced signal intensity variability (45, 46).

### 2.2. Pericardial disease

Symptomatic pericarditis is the major CMR-detectable abnormality affecting around 30% of patients of SLE (30) and 40% of patients of SS (47, 48). Chronic pericarditis is seen in around 70% of patients with SS (32). Asymptomatic pericardial involvement with autopsy confirmation has been reported in up to 80% of patients with SLE (3), about 50% of patients with MTCD (49), and 30% of Primary Sjogren's syndrome (50). However, tamponade occurs in less than 2% and constrictive pericarditis is extremely rare in patients with SLE (3).

CMR helps in the assessment of pericardial thickening, adhesions, and quantification of the severity of pericardial effusion, and may show signs of constrictive pericarditis even when the pericardial is not overly thick. Enhancement of pericardium on post-contrast images is the most useful diagnostic sign for the presence of pericarditis (51) (Figure 1). T1 mapping can differentiate exudative from transudative pericardial effusion using a predetermined threshold of 3,015 ms for pericardial effusion and 3,440 ms for pleural effusions with lower values suggesting the presence of exudates (52).

Exaggerated ventricular interdependence or ventricular coupling is unique to constrictive physiology and can be obviated on real-time cine CMR as an early diastolic septal inversion or flattening at the height of rapid deep inspiration, popularly known as septal bounce (53–55). In symptomatic patients with thickened pericardium, pericardial enhancement on LGE combined with either high signal intensity on T2w and/or elevated T1/T2 mapping indicates active inflammation that may necessitate anti-inflammatory therapy rather than surgery (56). T2 mapping of the pericardium is not ready for prime time as the pericardium being too thin walled it is difficult to avoid massive partial volume artefact.

### 2.3. Myocardial scar characterization on late gadolinium enhancement

**Figure 2:**
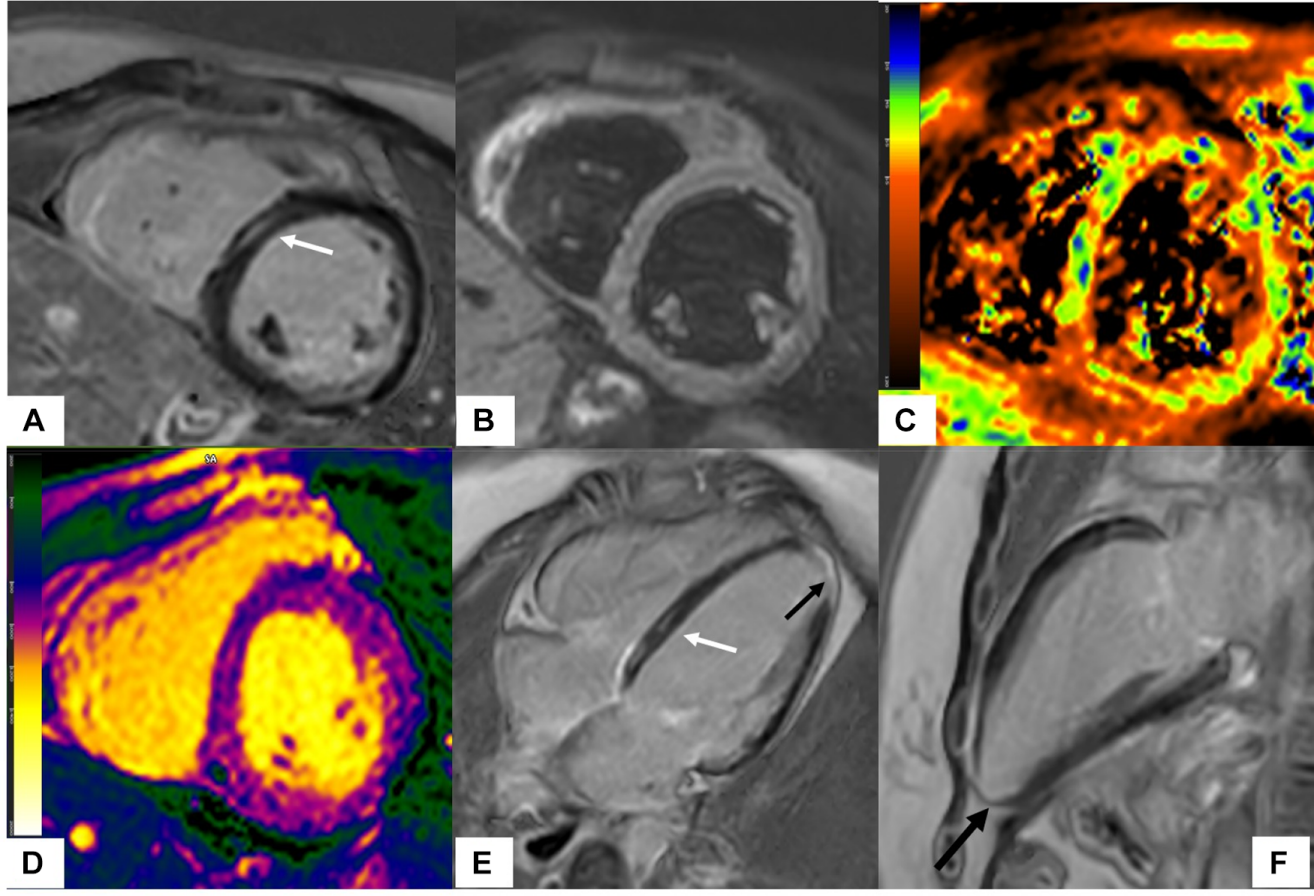
Scleroderma with mixed ischemic and non-ischemic cardiomyopathy on CMR. 20-year-old female with diffuse systemic sclerosis, ANA + anti Scl70 + with interstitial lung disease. Patient had occasional palpitations, ventricular tachycardia (VT) and premature ventricular contractions (PVC) burden of 13.2% on Holter monitoring. Cardiac MR (CMR) revealed normal size LV with low normal LV systolic function with left ventricular ejection fraction (LVEF) 54%, moderately dilated RV with moderately reduced right ventricular ejection fraction (RVEF) of 39%. On short-axis and 4-chamber late gadolinium enhancement phase-sensitive inversion recovery (LGE PSIR), mid myocardial late gadolinium enhancement was seen in the septum in a non-coronary distribution (**A,E** white arrows) without increased signal on short-axis T2w BB FS images (**B**), normal T2 values on T2 color map (**C**) and normal native T1 value on native T1 color map (**D**) These findings were suggestive of chronic myocardial fibrosis related to scleroderma involvement (42). Transmural LGE with akinetic apical anterior wall and apex was seen on the 4-chamber and 2-chamber views (black arrows **E,F**) suggestive of chronic microvascular injury and myocardial infarction in the absence of epicardial disease. Overall, findings of mixed ischemic and non-ischemic etiology were likely related to systemic sclerosis.

Patients with ARDs can develop myocardial replacement fibrosis due to infarction but also reactive fibrosis due to nonischemic insults. Ischemic cardiomyopathy is characterized by the presence of a subendocardial scar in a coronary distribution on LGE CMR. A nonischemic pattern of the scar on CMR includes diffuse subendocardial LGE in a non-coronary distribution, mid-myocardial or subepicardial pattern (Figure 2). Besides visual depiction of scar, CMR can provide scar quantification using different techniques on CMR such as full-width half maximum (FWHM), 5 standard deviations, or 6 standard deviations from remote myocardium (57). Acute and chronic myocardial processes can be characterized on CMR by combining information from LGE with T2 and T1 mapping sequences. T1/T2 mapping indices identify diffuse fibrosis and myocardial edema in patients with ARDs when significantly higher native T1 and T2 mapping values are present and they are independent of the presence of LGE (58).

Imaging within the first few minutes after contrast administration helps delineate microvascular obstruction which prevents contrast delivery to the infarct core and thus results in low signal on T1w imaging (59–61).

The use of T2w CMR in conjunction with LGE can help delineate the ischemic region at risk during acute myocardial infarction, which usually extends beyond the scar. It also helps evaluate the presence of coexistent myocardial inflammation in addition to scarring in patients with ARDs (10), Churg-Strauss syndrome (62), and SLE (39, 40). Rheumatic nonischemic lesions are mainly mid-myocardial or subepicardial, independent of the coronary distribution, and common at the inferior or inferolateral wall of LV thus mimicking the image pattern of viral myocarditis (63). The presence of edema on T2w mages in rheumatic patients is indicative of the acute phase of myocardial inflammation and can be identified simultaneously or early before the appearance of LGE lesions (38, 39). In the setting of myocarditis in SLE, the “age” of injury can be estimated on CMR with the presence of T2 and early gadolinium enhancement suggesting an acute process, and their normalization but persistence of LGE suggesting sub-acute or chronic stages of myocardial inflammation (38, 64, 65). The application of LGE CMR allows the detection of myocardial necrosis as reported in patients with vasculitis (10, 66) and SLE (67, 68).

Mavrogeni et al., characterized acute and chronic lesions as T2 signal intensity (SI) ratio >2 and positive LGE and T2 SI ratio <2 and positive LGE, respectively in patients with CTDs including sarcoidosis, SS, SLE, RA, and inflammatory myopathies. The etiology of myocardial injury was defined based on the distribution of CMR findings: vasculitis was defined when LGE was diffuse and subendocardial in distribution but not following coronary distribution, myocarditis was favored when LGE was subepicardial/intramural and myocardial infarction when LGE was subendocardial/transmural following a coronary territory (16). CMR pattern of diffuse subendocardial fibrosis on LGE is compatible with diffuse subendocardial vasculitis (DSV) caused by either diffuse myocardial ischemia due to vasculitis of small epicardial vessels or by granulomatous or eosinophilic infiltration (19, 20).

### 2.4. Myocardial ischemia

Abnormalities of myocardial perfusion can be commonly seen in ARDs. The presence of myocardial perfusion defects can also strongly and independently predict coronary artery disease in ARDs such as SLE (69–71). Coronary vasculitis with persistent inflammation, autoimmunity, immune complex deposition, and antiphospholipid antibodies are hypothesized to cause intimal damage followed by accelerated atherosclerosis in these patients. In the absence of epicardial coronary stenosis, an abnormal coronary flow reserve causes an impaired coronary microcirculation and is associated with a negative prognosis (72). Up to 40% of women with SLE with no history of coronary artery disease showed abnormal myocardial perfusion (66–68). Coronary dissection and coronary artery aneurysms may occur in patients with SLE (73). Accelerated or premature atherosclerotic disease is a leading cause of late death in patients with SLE (74–77). In SSc, coronary involvement is not a feature but coronary flow reserve may be lower when compared to healthy controls (78). Overt perfusion defects have been identified on stress CMR in SSc patients when there is a history of digital ulceration (79) and in patients with antiphospholipid syndrome (APS) without the presence of overt cardiac symptoms (80).

There is growing evidence in favor of CMR for evaluation of coronary artery disease in young women with ARD over SPECT due to lack of ionizing radiation and higher diagnostic accuracy as per the landmark MR-IMPACT impact (81, 82) and CE-MARC study studies (83). On CMR, myocardial Ischemia is defined by the presence of myocardial perfusion defects at stress during the first pass of gadolinium, which recovers at rest (84, 85). Presence of wall motion abnormalities, abnormal wall thickening, and perfusion defects on CMR during pharmacologic stress with dobutamine has improved sensitivity (86% vs. 74%) and specificity (86% vs. 70%) for the detection of myocardial ischemia over stress echo (86–88). Such a reliable assessment of subendocardial ischemia on CMR is made possible due to a higher spatial resolution of 2–3 mm. Recent advances in the CMR perfusion technique have made it possible to achieve a spatial resolution of around 1 mm in the imaging plane, especially at 3 T scanners (89–91). Quantitative assessment of subendocardial ischemia is also feasible on CMR using myocardial perfusion reserve index (MPRI) (92) and resting myocardial blood flow in hibernating segments (93) and these approaches are found superior to SPECT when detecting significant coronary stenosis by CMR (94). Newer technique of quantitative perfusion mapping is available at dedicated centers. This utilizes respiratory motion correction to generate perfusion maps with free breathing acquisition. Pixel-wise quantification of myocardial blood flow (MBF) at rest and during vasodilator-stress is possible at a high spatial resolution of approximately 2 mm that provides objective assessment of macro and microvascular ischemia (25).

Although acute myocardial infarction (MI) is a rather unusual event in rheumatic patients, the possibility of coexisting coronary artery disease with myocardial infarction should be always kept in mind during evaluation. Myocardial infarction from coronary microvascular disease exclusively presents as subendocardial lesions (95). Acute MI usually presents with concomitant myocardial edema that can be identified on T2w sequences. The presence of myocardial hemorrhage carries a poor prognosis and can be detected using T1w, T2w, and T2*w images (96).

### 2.5. Macro/microvasculopathy and vascular inflammation

The silent early phases of cardiovascular involvement in patients with ARD are generally due to the presence of microvascular disease. Chronic inflammation, the duration and activity of the autoimmune disease, and immunosuppressive therapy are specific risk factors for microvascular disease. Accelerated coronary atherosclerosis at a younger age than in the general population is due to the underlying presence of CVD. Unexplained arterial thrombosis and malignant arterial hypertension may also be seen in some patients with vasculitis (97).

There is potential for CMR angiography (CMRA) as an added imaging option when an assessment of vascular involvement is desired. It can be performed using gadolinium-enhanced MR angiogram or non-gadolinium-enhanced 3-dimensional SSFP sequences, particularly useful for the coronary arteries. CMR perfusion and mapping techniques can identify microvascular disease in patients with ARD (98), but CMRA is the technique of choice for the noninvasive evaluation of coronary arteries (aneurysms, ectasia) and great vessels (inflammation, aneurysms) that are frequently involved in systemic rheumatic diseases (60). Giant coronary artery aneurysms (CAA) can form from primary vasculitis, atherosclerosis, or a combination of both to infrequently cause acute coronary syndromes (ACS) in both SLE and rheumatoid arthritis RA or Rhupus syndrome (RhS), a rare entity characterized by overlapping rheumatoid arthritis (RA) and systemic lupus erythematosus (SLE) (99). Concomitant acquisition of CMR has the potential to mitigate additional costs to evaluate coronary arteries separately using CTA and reduce the burden of subjecting patients to multiple exams.

### 2.6. Valvular diseases

**Figure 3:**
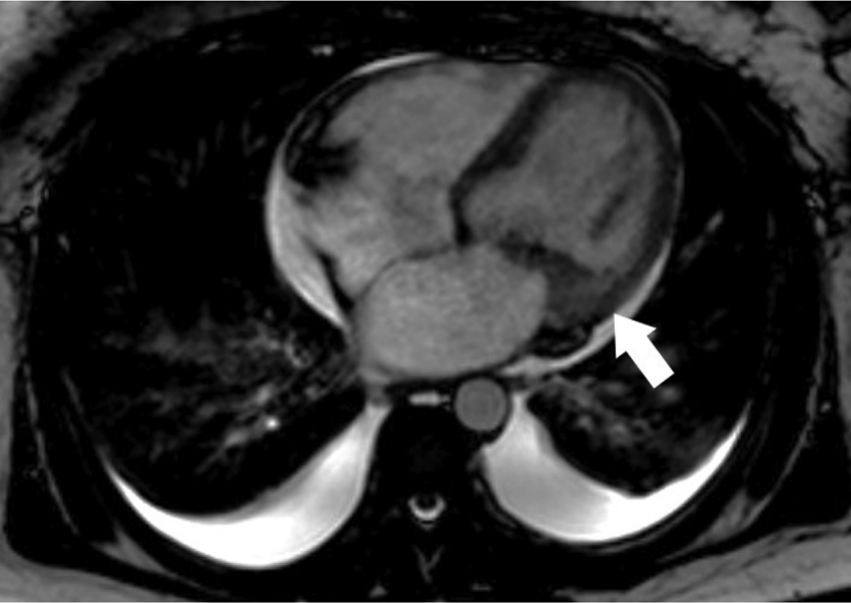
Libman-Sacks endocarditis on CMR. Cine 4-chamber view shows mass-like mitral valve vegetation and valve thickening (white arrow) consistent with Libman-Sacks endocarditis.

Libman-Sacks or marantic endocarditis consists of non-infective, verrucous valvular vegetation and is the most characteristic valve lesion in SLE (Figure 3). Most frequently, this involves the mitral valve and to a lesser degree, the aortic valve. Multi-valvular involvement can also occur in SLE. Roldan et al. described valvular abnormalities in 61% of 69 cases of SLE unrelated to disease severity, disease activity, or disease duration (100). An older prospective study of 132 consecutive patients reported valvular lesions in 22.7% of the cases associated with the presence of antiphospholipid antibodies (101). In SS, tricuspid regurgitation was seen in 40%, whereas thickening of the aortic valve and mitral valve leaflets in 12% and 8% respectively (45). Although echocardiography remains the modality of choice for evaluating valvular lesions, CMR can provide a highly reproducible serial assessment of valvular disease for the characterization of verrucous vegetation. CMR allows objective and reproducible quantification of mitral or tricuspid regurgitation and aortic valve assessment in patients with ARDs with low inter-study variability and operator dependence (102).

### 2.7. Pulmonary hypertension (PH)

Patients with ARDs often develop pulmonary hypertension during the natural course of the disease processes. Of all the ARDs, SS has the highest prevalence of pulmonary hypertension in up to half of the patients secondary to lung involvement, with a 1-year survival rate of only 50% (48, 103). The presence of PH in limited cutaneous SSc without concomitant interstitial lung disease results in an overall poor prognosis (32). Secondary pulmonary hypertension is seen in patients with Sjogren's syndrome (47) and is the leading cause of death in MTCD (104). Venous thrombo-embolic complications may cause pulmonary hypertension and right heart failure in SLE (29). The prevalence of asymptomatic pulmonary arterial hypertension in SLE was reported to be 10.8%, with a female-to-male ratio of 10:1 (105).

**Figure 4:**
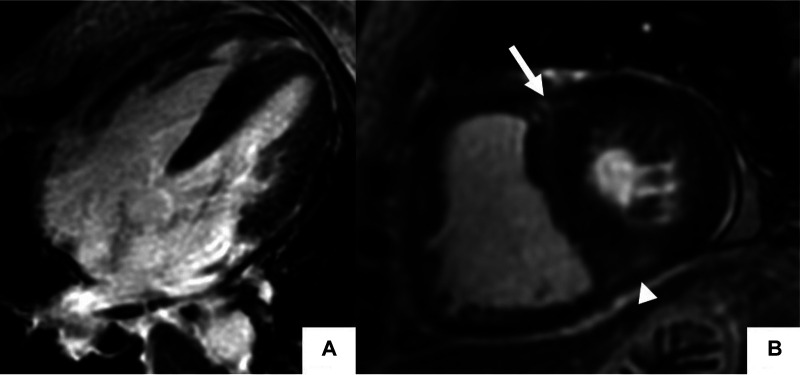
Late gadolinium enhancement cardiac MR (LGE-CMR) in polymyositis with pulmonary hypertension. Late gadolinium enhancement phase-sensitive inversion recovery (LGE-PSIR) images in the 4-chamber (**A**), and short-axis (**B**) showed increased RV and LV thickness with focal LGE at the superior (arrow) and inferior (arrowhead) RV insertion points in a patient with polymyositis with cardiac involvement.

Recently, the Pulmonary Vascular Research Institute recommended cardiac MRI to monitor right ventricular (RV) function in patients with pulmonary hypertension (106). On CMR, the most common but non-specific sign of PH is the presence of LGE at the RV insertion points, which is not related to disease severity (Figure 4). Diagnosis of pulmonary hypertension-related heart disease can be performed with CMR, but diagnosis of pulmonary hypertension alone has been challenging. A composite CMR model of interventricular septum angle (IVS), ventricular mass index (VMI), and black blood slow flow can be used for non-invasive diagnosis of PH with improved sensitivity (93%) and specificity (79%) with high correlation to mPAP (107). The interventricular septal (IVS) angle on CMR can help differentiate pre- and postcapillary PH from isolated postcapillary PH. An elevated systolic interventricular septal angle ≥ 160° in patients with combined pre- and postcapillary pulmonary hypertension can predict the presence of left-sided heart disease (108).

### 2.8. Diastolic or systolic heart failure

Excellent image quality and high spatial resolution achievable by CMR allow highly reproducible measurements of ventricular volumes and function (109). In SS, right heart failure is common and mostly secondary to lung fibrosis and pulmonary hypertension (44). LV impairment is the common endpoint and imaging biomarker in ARD and acute or chronic diffuse subendocardial vasculitis (DSV) (19).

LV diastolic dysfunction is seen in SS in 29% of the patients (45), and 42% of the cases of PM and DM (110). In limited SSc, subclinical LV dysfunction as evidenced by echocardiography and perfusion scintigraphy was found in 42% (32). In Sjogren's syndrome, diastolic dysfunction was found in 50% of evaluated patients and was noted to be independent of pericardial findings (111).

## 3. CMR signatures for risk stratification, prognostication, and guiding treatment strategies in ARDs

The development of clinically overt cardiac signs in patients with ARDs indicates advanced cardiac disease and carries a poor prognosis (112). Identification of disease acuity and various cardiovascular diseases process such as vasculitis, myocarditis, and myocardial infarction itself influences the CV risk stratification of ARD patients (12). Every morphologic or functional change in the myocardium, detected by any diagnostic technique, should motivate the early start of ACE inhibitors and b-blocker in patients with ARDs. Conventional and biologic disease-modifying antirheumatic drugs (DMARDs) have considerably improved long-term outcomes in ARDs not only by suppressing systemic inflammation but also by lowering the CVD burden (113–115). Improvement in inflammatory joint disease using anti-TNF-α therapies has also been associated with a reduction in cardiac events, further supporting the use of these disease-modifying agents (116, 117).

There are a lot of recent publications on how CMR can add value to risk stratification and the management of patients with ARDs. For example, the presence of late gadolinium enhancement (LGE) on CMR is related to a worse cardiovascular prognosis in these patients (118). Some studies suggest that edema/fibrosis visualization with CMR may have the potential to inform cardiac and rheumatic treatment modification in ARDs with or without abnormal routine cardiac evaluation.

Currently, there are no large multicenter trials to confirm the prognostic role of CMR described in smaller clinical trials and observational studies. However, early information provided by CMR, even in patients with normal systolic function, may be useful to change risk stratification and motivate the early start of cardiac medication (119).

CMR can help in the identification of occult myocardial lesions such as myocardial edema, myocarditis, diffuse subendocardial fibrosis, and subendocardial myocardial infarction that can be potentially reversed with appropriate management in treatment naïve ARDs (24). The recent clinical consensus document by EACVI advocates for the use of CMR for evaluating the effectiveness of immunomodulatory or cardioprotective treatment in patients with ARDs (23).

### 3.1. RA

Identification of myocarditis on CMR in RA is associated with a lower frequency of disease relapse and heart failure (18). When present, myocarditis, in contrast to pericarditis, is an indication for treatment with corticosteroids in RA (3). Minimal disease activity and short disease duration are associated with better outcomes in RA (13). The presence of myopericarditis in CMR can precede the development of relapse and subsequent congestive heart failure in patients with RA, a poor prognostic indicator in these patients (18).

Traditional DMARDs either as monotherapy or in combination with anti-TNF-α agents are extremely effective in RA (120). CMR-guided therapy had better outcomes in terms of a decrease in pericarditis recurrence and exposure to steroids in patients treated with colchicine and nonsteroidal anti-inflammatory drugs as the first-line therapy for acute pericarditis (48). In patients with RA, improvement in CFR without significant changes in plasma ADMA levels can help to monitor the effect of anti-rheumatic therapy with DMARD or anti-TNFα treatment on endothelial dysfunction and disease activity (2). These parameters can be tracked using perfusion CMR for treatment monitoring in these patients. The administration of tocilizumab (TCZ) is associated with reduced RA disease activity and improved LV function as assessed by CMR (121). Additionally, TCZ can improve LVEF and cause a decline in LV mass index which is associated with a decrease in disease activity (122).

### 3.2. SLE

Identifying subclinical myocardial involvement in CMR can add prognostic value in SLE as cardiovascular complications are a leading cause of death in these patients, but clinical signs may be identified in <10% of patients (30). In autopsy studies, myocarditis was noted in up to 30%–50% of lupus patients (31, 123), so it is a common subclinical feature of the disease. Accordingly, T2-mapping techniques can frequently identify increased myocardial T2 in SLE patients with subclinical myocardial edema (124). Therefore, low-grade myocardial inflammation may be detected on CMR in SLE patients with quiescent disease and normal cardiac function, who may benefit from the initiation of immunosuppressive treatment.

Interestingly, the introduction of corticosteroids to SLE management has decreased the incidence of autopsy-identified lupus myocarditis from 50%–75% (31, 125) to about 25%–30% (123, 126). Lupus myocarditis presenting as cardiogenic shock may require mechanical support and other drugs such as rituximab, intravenous immunoglobulin azathioprine, and cyclophosphamide (127). CMR can help in assessing accurate improvement of left ventricle function and response to steroids in such patients.

Significantly higher inflammatory markers, LV mass, native T1 and T2 values, and reduced longitudinal strain has been reported in patients with SLE. Greater improvement in native T1 and T2 values in patients after intensified anti-inflammatory treatment suggests a role of native T1 and T2 as significant predictors of treatment response on follow-up CMR (128).

### 3.3. Scleroderma

**Figure 5:**
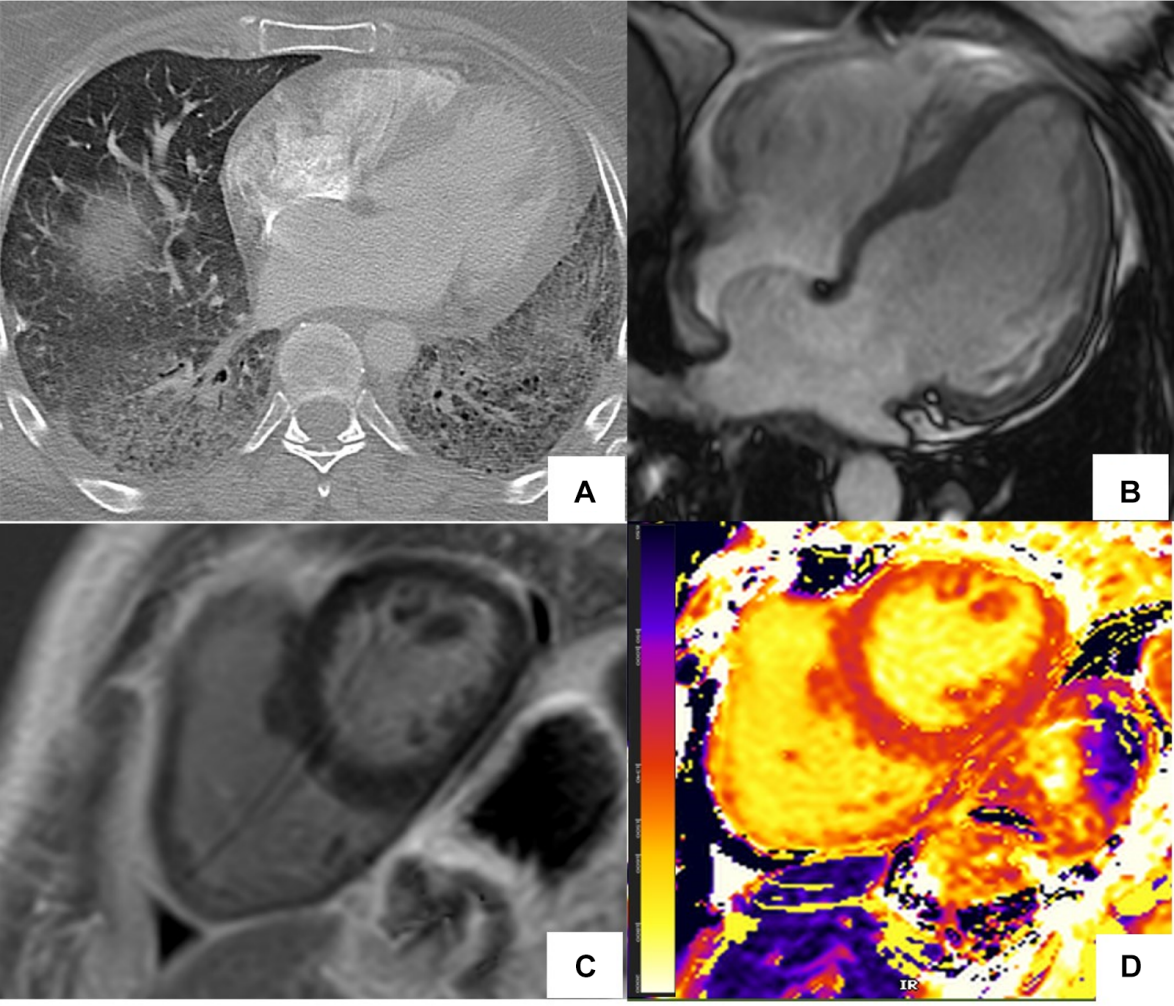
Abnormal cardiac MR (CMR) T1 mapping in subclinical myocardial inflammation in a patient with scleroderma. 37-year-old female with scleroderma-related interstitial lung disease (**A** Chest CT) and increasing dyspnea on exertion. 1.5 T cine CMR had a normal LV ejection fraction of 59% and no regional wall motion abnormality. 4ch cine showed flattening of the inter-ventricular septum consistent with known pulmonary hypertension (**B**) On short-axis late gadolinium enhancement phase-sensitive inversion recovery (LGE-PSIR) there was no myocardial late gadolinium enhancement, only trace pericardial effusion (**C**) Native T1 values were elevated to 1,250 ms on the native T1 color map (**D**) suggesting active myocardial inflammation.

Myocarditis is a common finding in SSc with recent-onset cardiac involvement and its early diagnosis allows timely immunosuppressive treatment leading to the prevention of cardiac damage in most cases (129) (Figure 5). Rest CMR perfusion index can be used to monitor patients with scleroderma with early myocardial perfusion defects on treatment with Nifedipine, a calcium antagonist. A significant increase in the MRI perfusion index was reported after 14 days of oral treatment with Nifedipine 60 mg/day with a mean perfusion index of 0.26 v 0.19 at baseline, *p* = 0.0003. Improvements were also noted in systolic and diastolic strain rates on TTE post-therapy thus implicating the utility of CMR strain in the follow-up of such patients (130).

Stress CMR can detect silent myocardial Raynaud phenomena (RP) in patients with connective tissue diseases (CTD). Myocardial perfusion reserve index (MPRI) reduction is common in both primary and secondary RP, but more severe in secondary RP irrespective of treatment with calcium channel blockers. Identification of abnormal MPRI can help with the early initiation of treatment pharmacological treatment (131).

### 3.4. Vasculitis

CMR can help with treatment monitoring and temporal changes in acute or chronic diffuse subendocardial vasculitis (DSV). In a small study of 20 patients with ARD, CMR detected DSV had a rapid clinical improvement after treatment modification with the resolution of CMR findings on 75% of patients. In chronic DSV, clinical improvement was seen after the initiation of ACE inhibitors but there were no changes in CMR findings of DSV on follow-up imaging. CMR also identified improvement of LV dysfunction in most acute and some chronic DSV. However, those with chronic DSV never achieved normal LV function (19). In chronic cases, identifying DSV to help with an early start of ACE inhibitors even in those with normal LVEF may help prevent overt LV dysfunction (132).

### 3.5. ARD-related PAH

PAH may complicate various ARDs such as SSc, SLE, MCTD, and RA. Novel targeted therapies have demonstrated a trend toward improvement of outcomes in ARD-related PAH (133–135). CMR volumetric and functional indices and native T1 mapping help with the assessment of treatment response and risk stratification when incorporated into PH risk scores, and carry prognostic value in PH. The prognostic features in patients with connective tissue disease (CTD) are found to be different from other PAH subgroups with metrics such as ventricular-vascular coupling (ratio of end-systolic to arterial elastances i.e., Ees/Ea) and RV mass being more significant than RV function and volume (136). The European Society of Cardiology and European Respiratory Society and the REVEAL (North American Registry to Evaluate Early and Long-Term PAH Disease Management) risk score calculator (REVEAL 2.0) identified CMR-based percentage-predicted right ventricular end-systolic volume index (RV ESVi) can improve risk stratification in PH when used in conjunction with current risk stratification approaches. RV ESVi threshold of 227% or a left ventricular end-diastolic volume index of 58 ml/m^2^ on CMR identified patients at high risk of 1-year mortality (137).

RV ejection fraction was the strongest and most well-established predictor of mortality in PAH in eight studies (539 patients) that investigated 21 different CMR findings. In addition, increased RV volumes and decreased LV end-diastolic volume at baseline have been associated with a higher mortality risk in PAH patients (138). Furthermore, because of lower measurement variability, ejection fraction derived from CMR is more cost-effective in PAH medication trials than echocardiography (139).

Other CMR features can be used for prognostic characterization in patients with PH. The myocardial strain has emerged as an early marker for biventricular dysfunction as well as a predictor for mortality (140). An elevated systolic interventricular septal angle ≥160° in patients with combined pre- and postcapillary pulmonary hypertension is predictive of left-sided disease and a poor prognostic indicator with an increased risk of death (103). Increased trabeculation at the marginal IVS on CMR is found to be associated with severe PH, reduced RV ejection fraction (RVEF), and exercise tolerance (141).

CMR can help in monitoring response to conventional and newer therapies for PH. The effect of sildenafil in addition to conventional treatment of PAH in patients with SLE and SSc has been previously evaluated with CMR. It has been shown that the combination treatment reduced the RV mass and improved cardiac function and exercise capacity in patients with PAH, WHO functional class III (142). CMR can be used to assess the response to newer combination therapy with Ambrisentan and Tadalafil by measuring improved RV contractility and function-reduced RV mass in CTD PAH such as in treatment-naive patients with SSc–PAH (143, 144).

## 4. CMR to identify cardiotoxicity related to immunomodulation therapies

**Figure 6:**
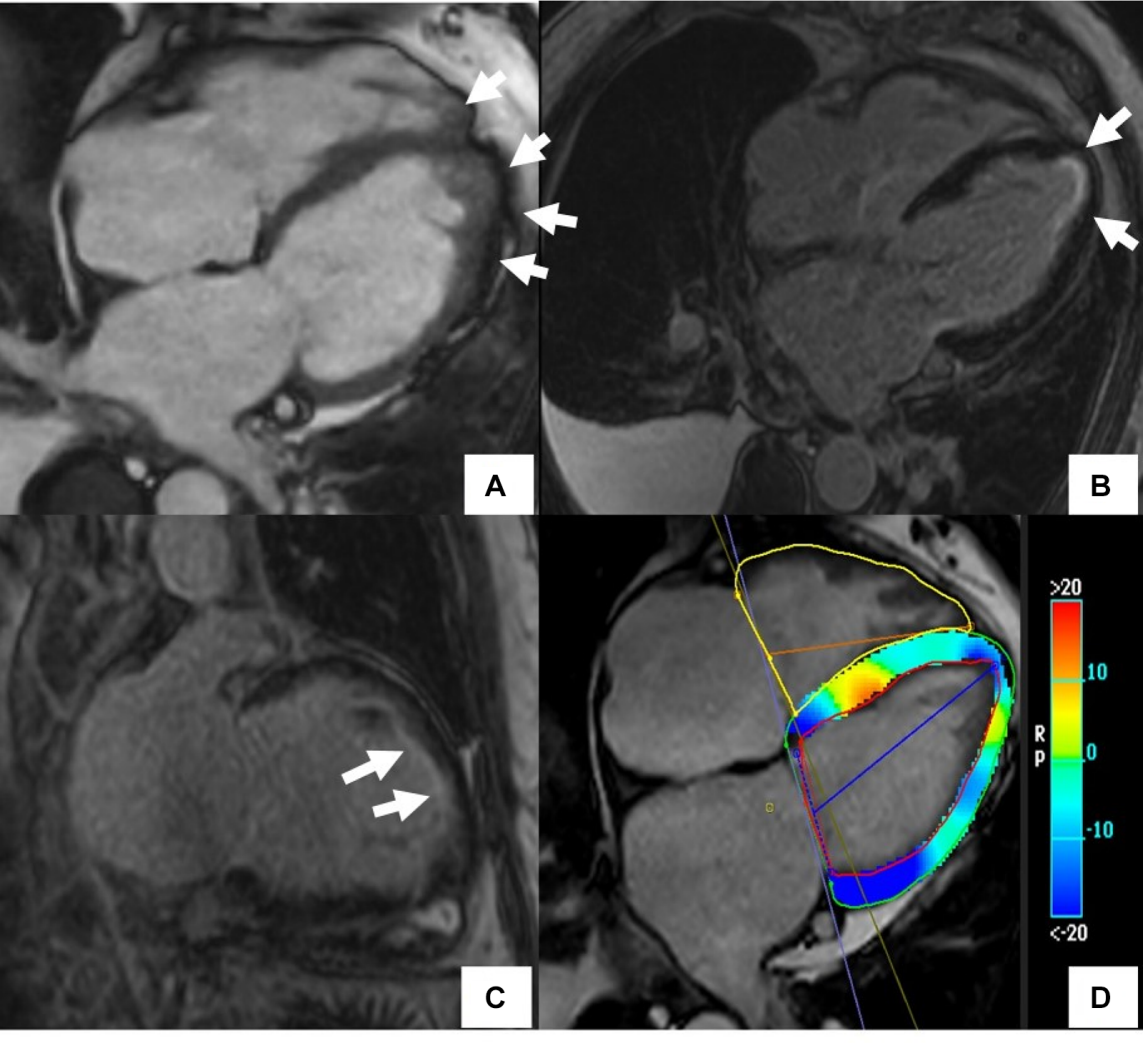
Cardiac MR (CMR) findings in a patient with drug-induced cardiotoxicity. The cine image in the 4-chamber plane (**A**) showed hyper-trabeculations in LV and RV apex (arrows). Late gadolinium enhancement phase-sensitive inversion recovery (LGE-PSIR) images showed diffuse subendocardial fibrosis (arrows) (**B,C**). There was also abnormal longitudinal strain relatively preserving the base on the feature tracking color map (**D**).

Cardiotoxicity occurs mainly from polypharmacy, overdosage, and long pharmacotherapy with conventional disease-modifying antirheumatic drugs (DMARD) and biologic agents such as Tumor Necrosis Factor (TNF)-α blockers and IL-6 receptor Inhibitors (145–147). Acute and chronic adverse events include infusion-related hypertension, myocardial ischemia, myopericarditis, restrictive cardiomyopathy (Figure 6), and congestive heart failure (148). CMR has proven clinical utility in identifying cardiotoxicity caused by immune checkpoint inhibitors (ICI) (149). A study on 104 patients with ICI-associated myocarditis showed frequent CMR abnormalities correlating with histological findings, including LGE in areas of replacement fibrosis and T2 abnormalities in lymphocytic infiltration (150).

Chronic use of hydroxyl-chloroquine may result in acquired lysosomal storage disorder, leading to drug-induced cardiomyopathy characterized by concentric hypertrophy on CMR and rhythm disturbances on ECG (151). CMR can also be used to establish treatment responses in patients with extracardiac symptoms when there is a secondary improvement in cardiac imaging biomarkers (119).

## 5. Limitations of CMR, recent advances, current research gaps, and potential developments in the field

One of the limitations of CMR is related to the minimal risk of developing nephrogenic systemic fibrosis (NSF), particularly in patients with renal dysfunction, typically with a glomerular filtration rate of <30 ml/min/1.73 m^2^ and/or patients on hemodialysis (152). However the risk of NSF is very low with macrocyclic contrast agents estimated at 0.7%. There is a theoretical risk of brain accumulation of GBCA that has been discussed in the literature (153). Other limitations include a lack of cost-effectiveness analysis of CMR use in patients with ARDs. Finally, CMR is contra-indicated in patients with metal fragments in the eyes and may require special safety protocols in patients with devices that are not MR-conditional (154).

Research gaps exist in at least three levels of evidence to document the necessity of additive anti-rheumatic treatment in ARD patients with CMR evidence of myocardial inflammation; observational data derived from imaging registries with adequate phenotype, treatment, and outcome data is not available; longitudinal and long-term CMR-based observational studies monitoring ARD patients on antirheumatic medication is lacking; randomized controlled trials of antirheumatic treatment based informed by CMR findings alone, with long-term outcomes, are needed (155).

Advanced CMR applications have the potential to characterize the entire spectrum of cardiovascular involvement and provide a one-stop shop for patients with ARDs. Currently, there is limited data on the utility of advanced imaging techniques such as texture analysis, strain, and 4D flow assessment in patients with ARDs. Recent studies show the advantage of radiomics-based texture analysis of T1 and T2 mapping to define infarct-like myocarditis with high sensitivity and specificity (156). Biventricular strain is found to be significantly impaired in PH and allows early detection of right and left heart dysfunction (157) and in patients with cardiotoxicity (158) (Figure 6). 4D- flow CMR is being investigated for its ability to identify abnormal flow patterns in the main pulmonary artery (MPA) and its association with PH. A recent study showed the potential for 4D-flow CMR to estimate mean PAP (mPAP), track PVR changes, MPA wall shear stress (WSS), and quantification of the severity of tricuspid regurgitation in pulmonary arterial hypertension and PH due to left heart disease, chronic lung disease or chronic thrombo-emboli (CTE-PH) (159).

Potential areas of research identified include the need for larger multicenter observational or interventional trials to validate CMR prognostic markers in ARDs. There are also few studies with a head-to-head comparison of CMR diagnostic performance compared to routinely used cardiovascular imaging modalities or endomyocardial biopsy for diagnosis. Other areas of potential research include the assessment of the utility of CMR in monitoring response to immunomodulatory and cardioprotective medications and targeted therapies for myocardial fibrosis (160). Exploratory studies designed specifically to identify associations between CMR biomarkers and circulating levels of inflammatory biomarkers and ARD-associated autoantibodies are needed. Finally, prospective studies on the integration of CMR with electroanatomic mapping to identify sites of the arrhythmogenic substrate to guide ablations and improve outcomes are desirable (161).

## Conclusion

CMR has the ability to identify cardiovascular abnormalities in patients with ARDs and can be used as a tool for the early detection of disease in asymptomatic patients. CMR is a promising method for the prognostic characterization of these patients and may be used to identify patients that could benefit from pharmacological interventions. Finally, CMR is a robust tool that could add value to monitoring treatment response and tailoring treatment strategies for patients with ARDs and cardiovascular involvement.
